# Editorialets

**Published:** 1908-05

**Authors:** 


### Editorialets.

American Medical Editors' Association.—The annual meeting of this society will be held at the Auditorium Hotel, Chicago, on May 30th and June 1st. An extensive and interesting program has been prepared, and every member of the Association is urged to be present, and editors of medical magazines, not now affiliated with this society, are also invited to meet with them. Do not forget the date, Saturday, May 30th, and Monday, June 1st.

---

Dr. S. M. Jenkins and Miss Shirley Hassler, of Enid, Oklahoma, were married April 29th ult.

---

Mrs. Hons, wife of Dr. J. M. Hons, of San Marcos, Texas, died at their home April 26th (ult.), after a protracted illness.

---

Dr. J. R. Rucker, an old practician of medicine, died at his home at Temple, Texas, April 19th ult., aged 55. He was a brother-in-law of Senator Bailey.

---

Falling in Love is the beginning of all wisdom, all sympathy, all compassion, all art, all religion; and in its larger sense is the one thing in life worth doing.—*The Fra.*

---

Dr. Wallace Rouse, a lecturer in the Medical Department, University of Texas, Galveston, while fishing from the pier out on the government jetties at the mouth of Galveston Bay, was struck by lightning and instantly killed April 21st ult. The lightning struck his steel fishing rod. Several of the party were stunned, but not seriously hurt. Dr. Rouse was 36 years of age and a graduate of the Medical Department, University of Texas, 1902.

---

The *Charlotte Medical Journal* and the *Carolina Medical Journal* have been consolidated. A stock company has been created which will conduct one journal in the future. The journal of the new corporation will be known as the *Charlotte Medical Journal*, and will retain the same architectural features, business and editorial management as the present *Charlotte Medical Journal*. The *Journal*, with its new influences, will be enlarged and, in many respects, greatly improved. It will remain a typical Southern journal, and will be devoted to the best interests of each member of the medical profession of the South. Dr. E. C. Register is editor.

---

What They Say: I called on my subscribers to *ante,* as the P. O. Department has ruled that I shall drop all who don't do so, and they just rattled the checks in,—most of them with kind words, for which I earnestly thank them. I can count on my "old guard" in a tight. Here are extracts from a few letters received in response to my appeal:

I regard the "Red Back" as my old friend—one with whom I can differ regarding certain policies, and yet feel that it is as honest as I am in its opinions. May you live long and prosper.

Always as your friend,

M. B. Grace, Seguin, Texas.

---

It is read with pleasure by my wife as well as by myself. Wishing you and the good "Red Back" continued success, I am,

Very truly yours,

H. A. Fee, M. D., San Antonio.

---

Rusk, Texas, April 11, 1908.

I would be lonesome without the "Red Back." [Twenty-one years.]

A. H. McCord,
State Prison Physician.

---

Galveston, Texas, April 13, 1908.

*Dr. F. E. Daniel, Austin, Texas.*

My Dear Doctor: Enclosed please find $5. "Now will you be good?" Your friend,

A. W. Fly.

[Five years in advance, 1913.]

---

Winnsboro, Texas, April 8, 1908.

*Dr. Daniel.*

Dear Sir: Please find $1 for subscription to "Red Back." Wishing you a long (yet) and useful life, and may success ever be with you, I remain, as ever, Your friend,

T. N. Skeen.

---

Lavernia, Texas, April, 13, 1908.

*Dr. F. E. Daniel, Austin, Texas.*

Dear Doctor: Please find enclosed P. O. money for $2, which will pay for my subscription to the "Red Back" for this year and next. The "Red Back" is worth many times its price and I welcome it always. Too good to do without.

Fraternally yours,

R. G. Martin, M. D.

WACO, TEXAS, April 22, 1908.

*Dear Doctor Daniel:*

Can't do without the "Red Back" and never expect to try to do so while you are the editor.

J. T. HARRINGTON.

---

DAYTON, TEXAS, April 9, 1908.

*Dr. F. E. Daniel, Austin, Texas.*

DEAR DOCTOR: Enclosed you will please find my personal check for $2. Let the "Red Back" come and notify me when my subscription expires. Fraternally,

J. T. TADLOCK, M. D.

---

MONROE, LA., April 6, 1908.

*Dr. F. E. Daniel, Austin, Texas.*

MY DEAR DOCTOR: The "Red Back" came to me this morning. I read your notice on page 413, and hasten to you a check. I do not want to be among those who help to break you. I am always interested in the "Red Back." It is like getting a letter from home. With best wishes to you, I am,

Cordially yours,

W. E. PUGH.

---

TEXARKANA, TEXAS, April 11, 1908.

*Dr. F. E. Daniel, Austin, Texas.*

MY DEAR DOCTOR: Enclosed find exchange for $1 in payment of subscription for JOURNAL up to June, 1908. I think I subscribed for the JOURNAL twenty-three years ago, and have enjoyed each number. I am now in my 72d year, having practiced medicine fifty years. It is now time for me to retire from an active practice, and will ask you to discontinue the JOURNAL when my subscription expires. [Not much. I will send it complimentary as long as we live.] "Ere I take the first step on that narrow isthmus that separates time from eternity," I wish to acknowledge my appreciation of yourself and the JOURNAL. May God bless you is my prayer.

R. W. READ.

---

And here is what Sharp & Dohme say:

BALTIMORE, April 17, 1908.

*The Texas Medical Journal, Austin, Texas.*

GENTLEMEN: It is purely a formal matter, but always a delightful business procedure to renew our arrangements with the TEXAS MEDICAL JOURNAL. Please consider this as a formal renewal of the old arrangement for the same space we have occupied for *more than twenty years,* and which we hope to be able to occupy for *another double decade.*

Taking this opportunity to extend our most cordial greetings to your perennially youthful and always gracious editor, and hoping that Mrs. Daniel enjoys excellent health, we beg to remain,

Sincerely yours,

SHARP & DOHME.

